# Supplementary material for: Complex‐centric proteome profiling by SEC‐SWATH‐MS
Source: Mol Syst Biol. 2019 Jan 14;15(1):e8438. doi: 10.15252/msb.20188438 (PMC6346213; doi:10.15252/msb.20188438)
Supplement: Supplementary file 8 — Dataset EV7 [file MSB-15-e8438-s008.zip › feature_plots_string/P62851.pdf]

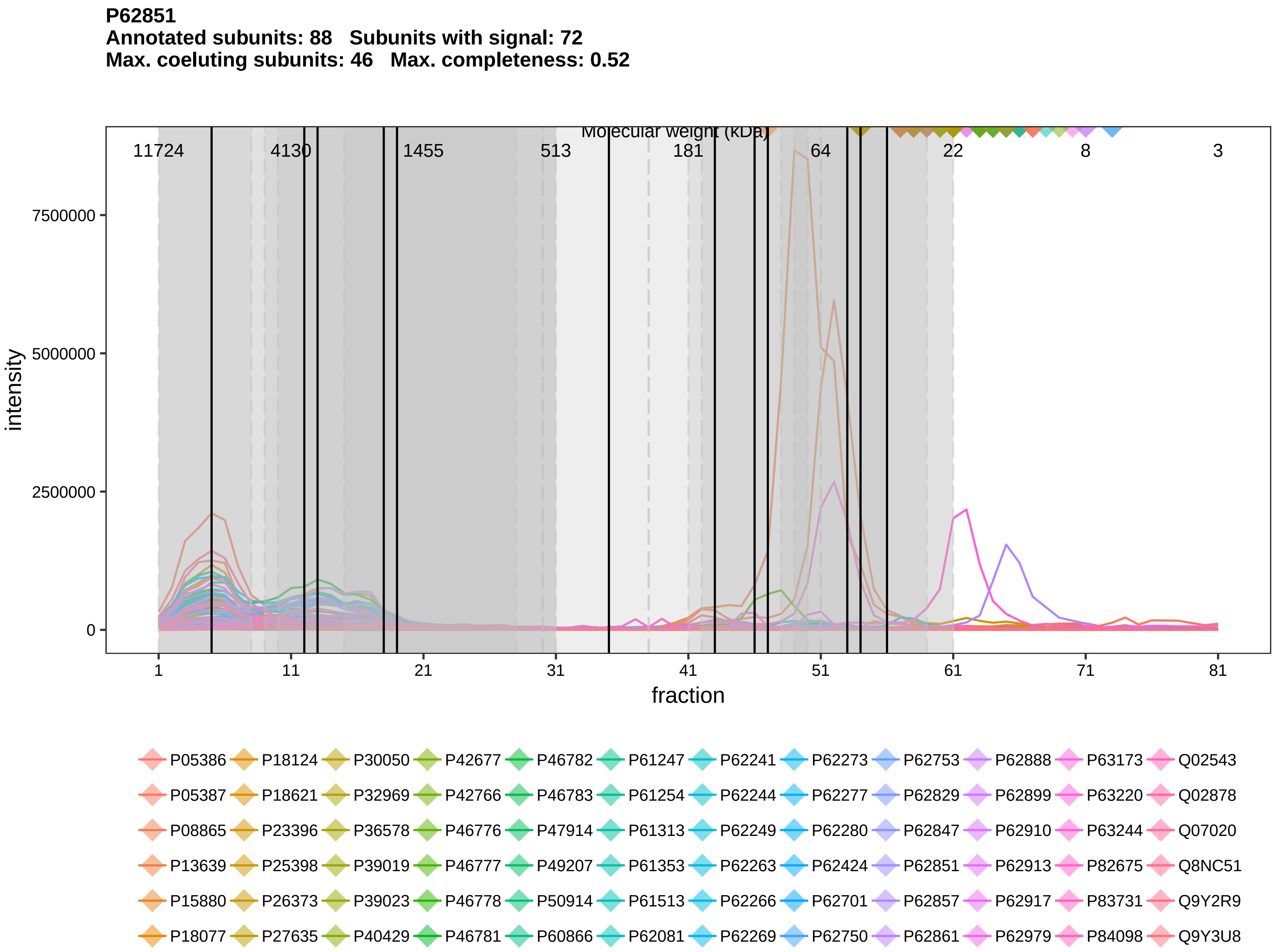
P62851
Annotated subunits: 88   Subunits with signal: 72
Max. coeluting subunits: 46   Max. completeness: 0.52
Molecular weight (kDa)
11724
4130
1455
513
181
64
22
8
3
intensity
7500000
5000000
2500000
0
1
11
21
31
41
51
61
71
81
fraction
P05386
P05387
P08865
P13639
P15880
P18077
P18124
P18621
P23396
P25398
P26373
P27635
P30050
P32969
P36578
P39019
P39023
P40429
P42677
P42766
P46776
P46777
P46778
P46781
P46782
P46783
P47914
P49207
P50914
P60866
P61247
P61254
P61313
P61353
P61513
P62081
P62241
P62244
P62249
P62263
P62266
P62269
P62273
P62277
P62280
P62424
P62701
P62750
P62753
P62829
P62847
P62851
P62857
P62861
P62888
P62899
P62910
P62913
P62917
P62979
P63173
P63220
P63244
P82675
P83731
P84098
Q02543
Q02878
Q07020
Q8NC51
Q9Y2R9
Q9Y3U8
